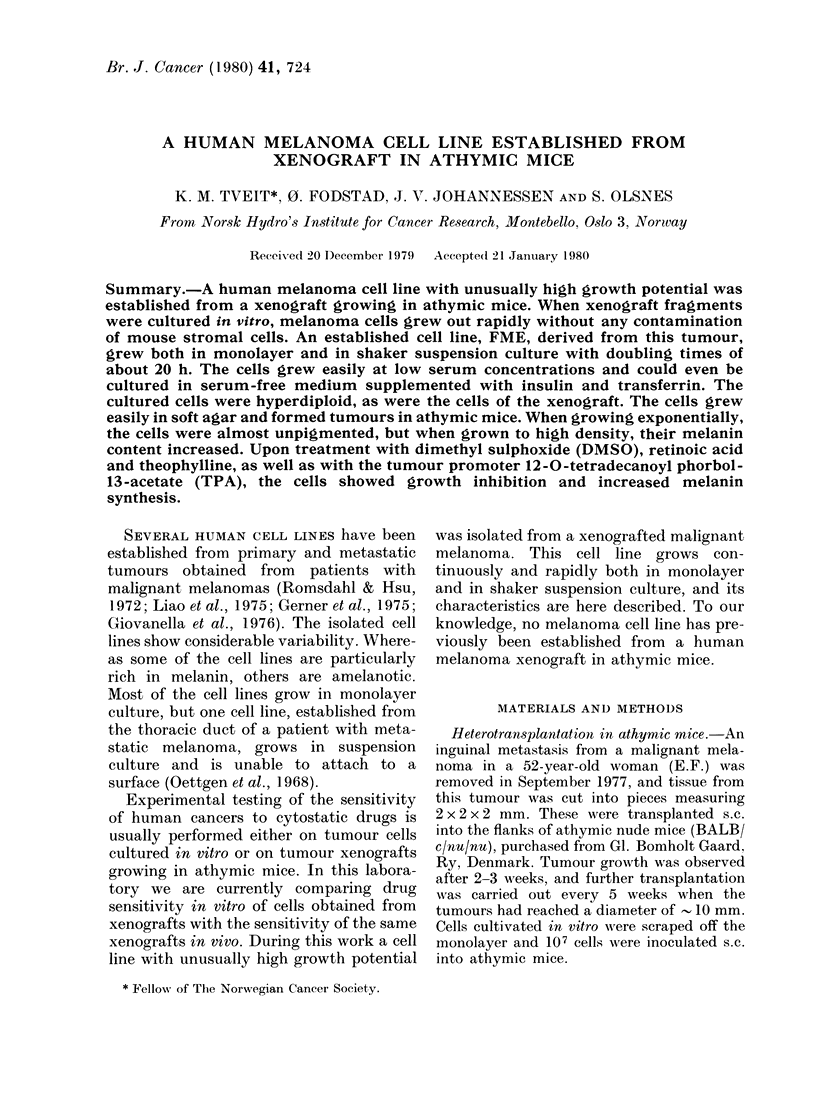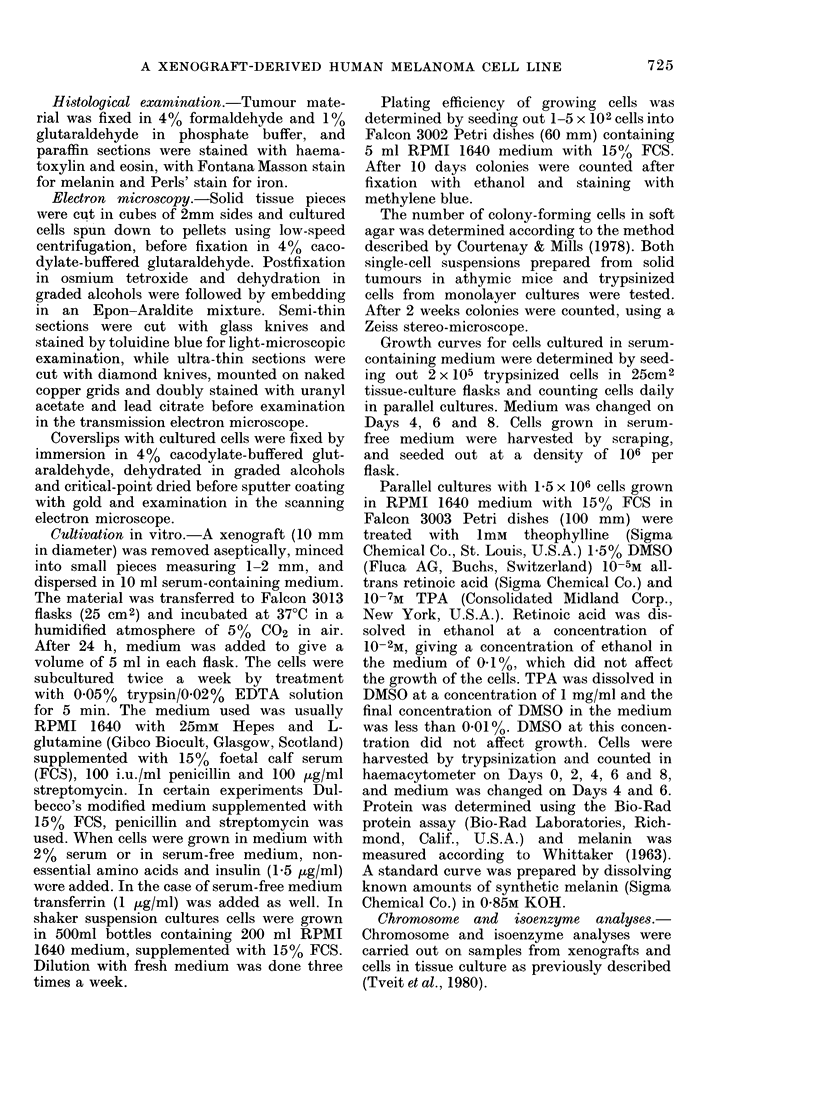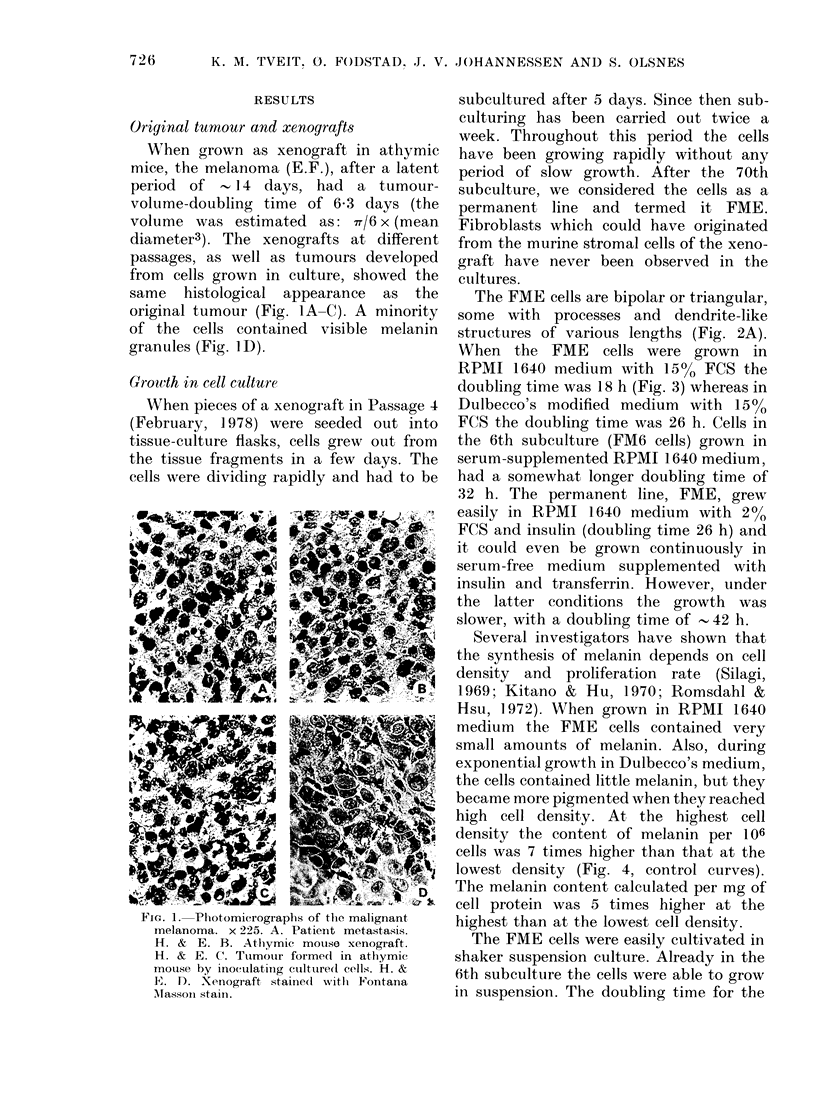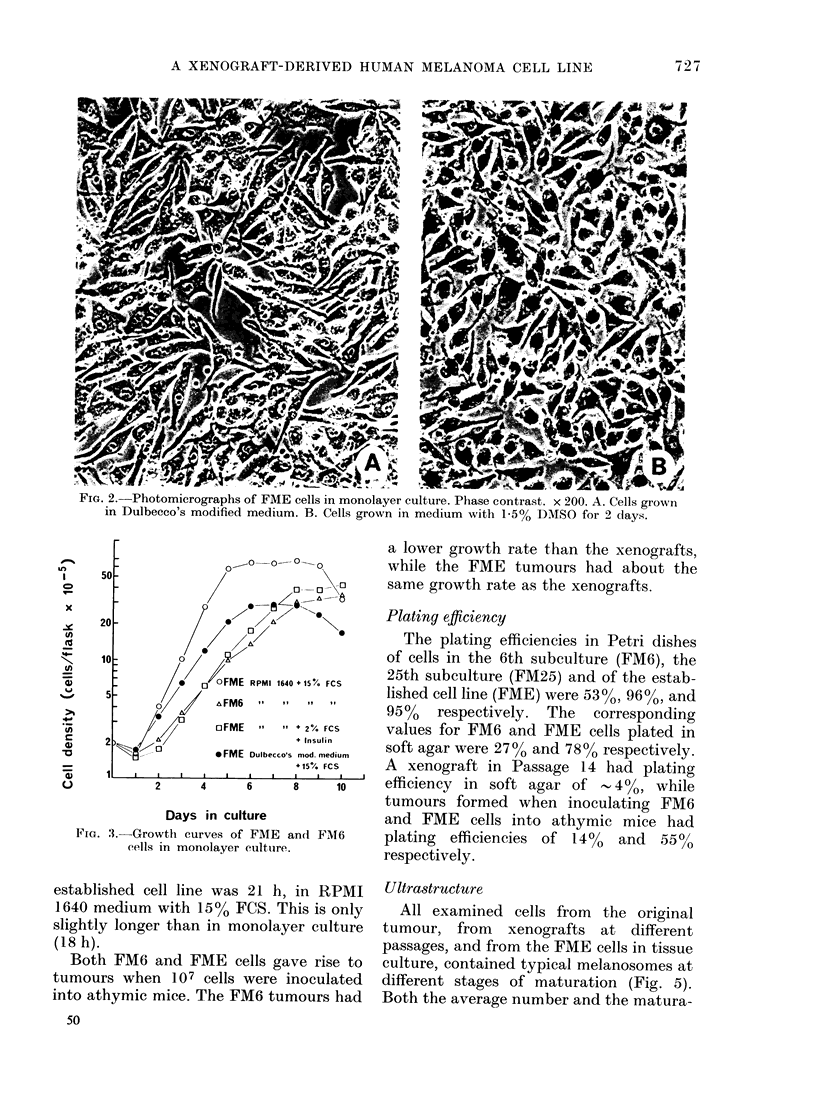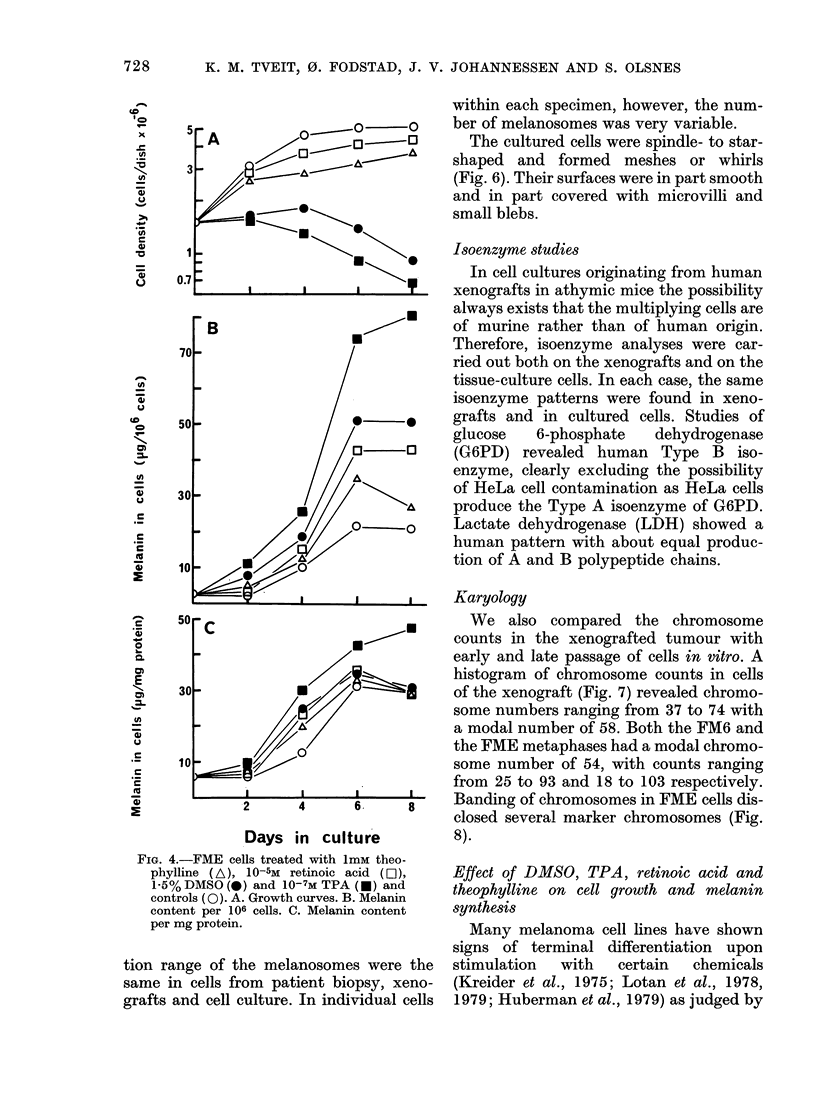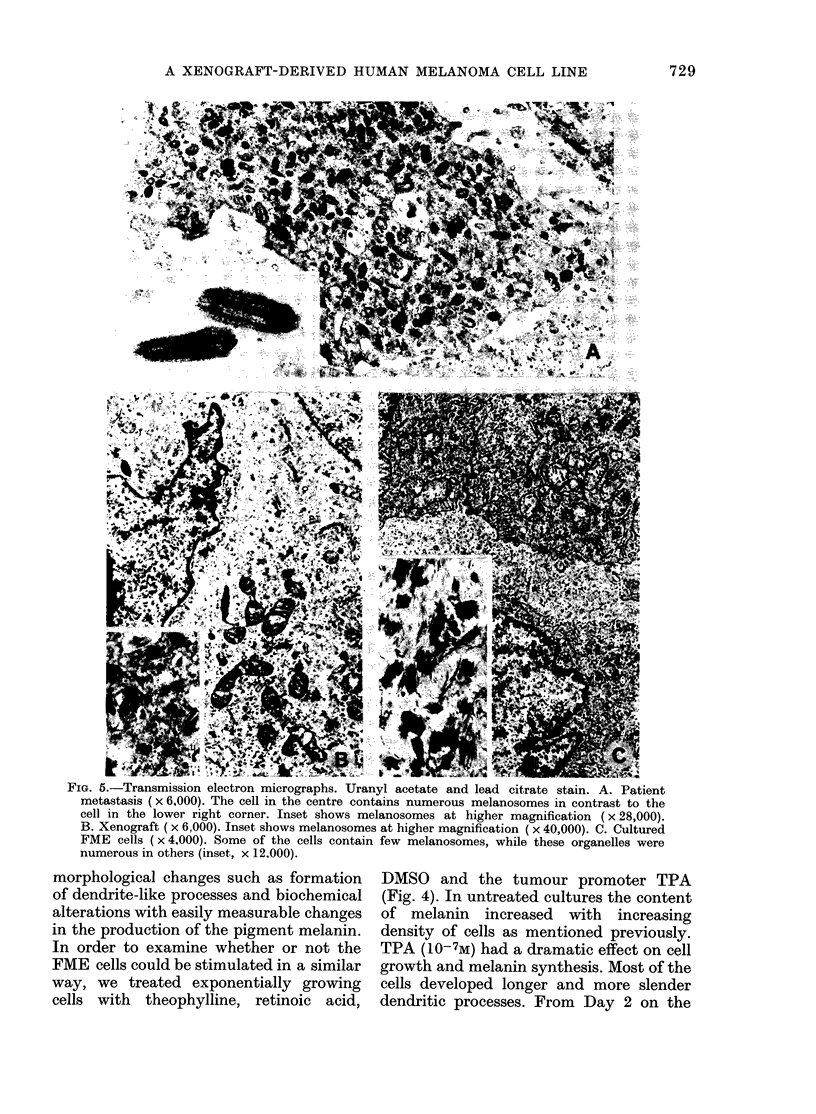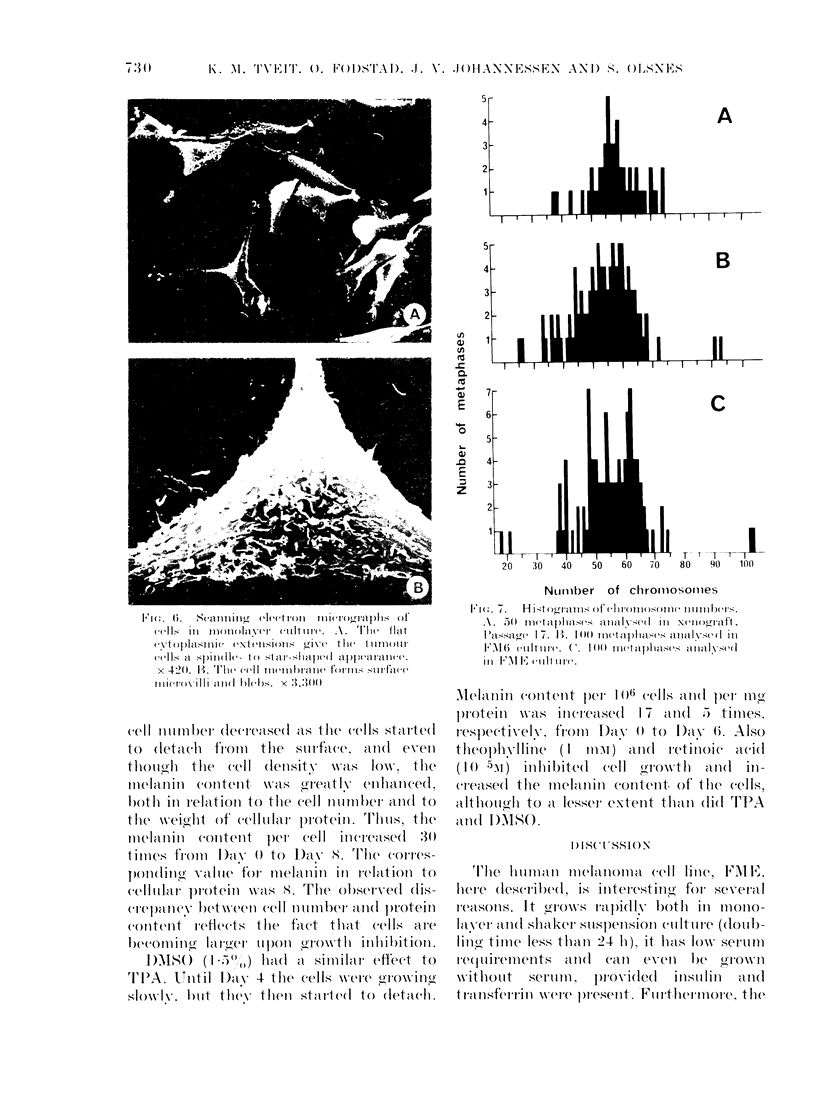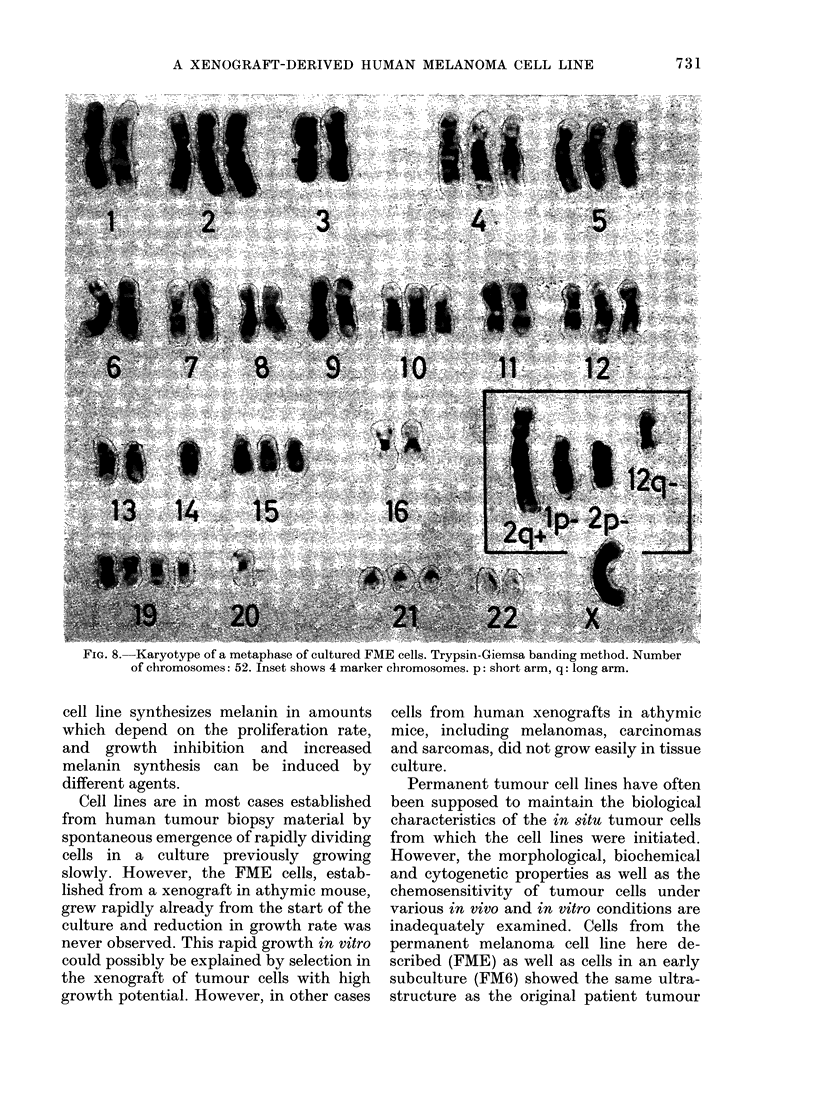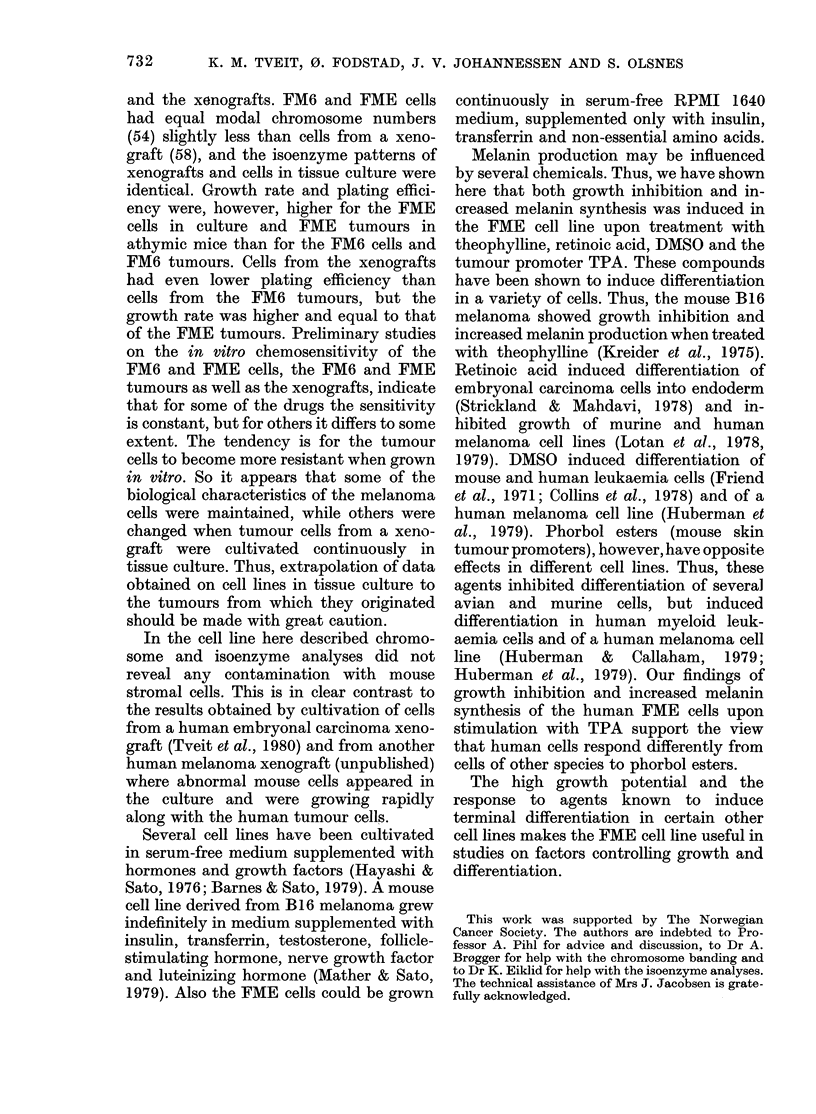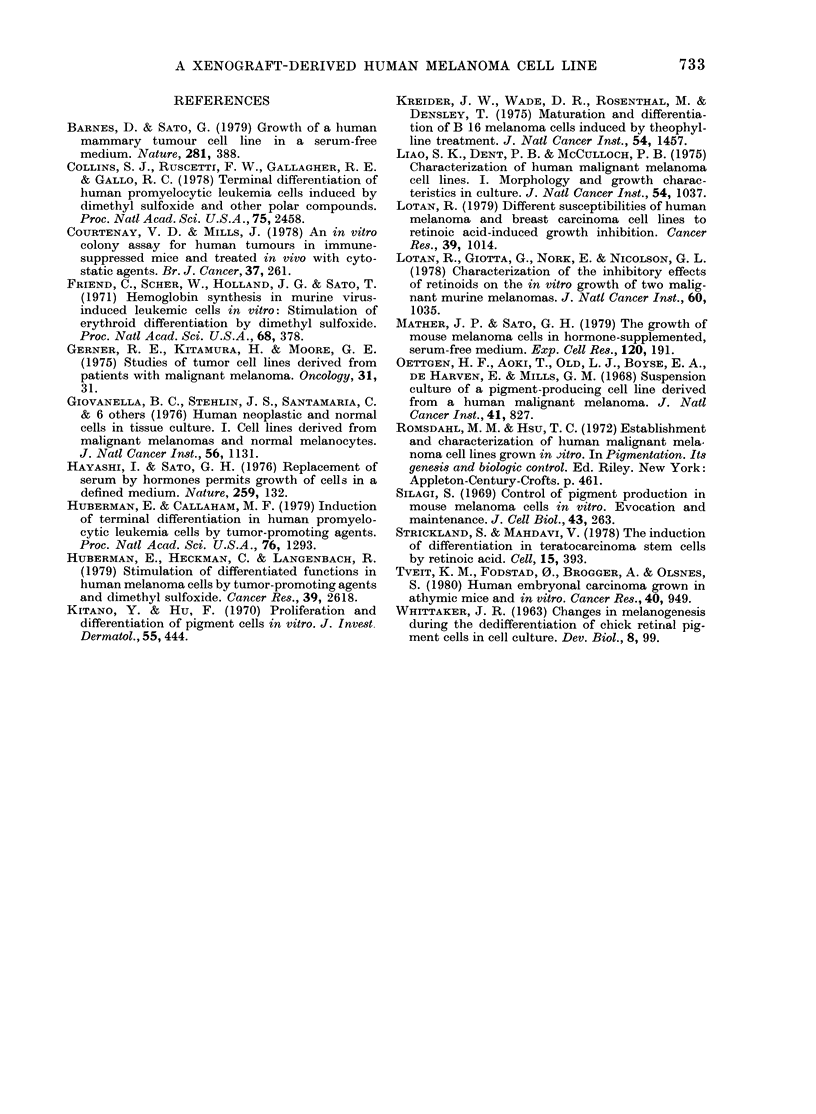# A human melanoma cell line established from xenograft in athymic mice.

**DOI:** 10.1038/bjc.1980.134

**Published:** 1980-05

**Authors:** K. M. Tveit, O. Fodstad, J. V. Johannessen, S. Olsnes

## Abstract

**Images:**


*Br. J. Cancer* (1980) **41**, 724

### A HUMAN MELANOMA CELL LINE ESTABLISHED FROM XENOGRAFT IN ATHYMIC MICE

K. M. TVEIT*, Ø. FODSTAD, J. V. JOHANNESSEN AND S. OLSNES

*From Norsk Hydro's Institute for Cancer Research, Montebello, Oslo 3, Norway*

Received 20 December 1979 Accepted 21 January 1980

**Summary.—A human melanoma cell line with unusually high growth potential was established from a xenograft growing in athymic mice. When xenograft fragments were cultured *in vitro*, melanoma cells grew out rapidly without any contamination of mouse stromal cells. An established cell line, FME, derived from this tumour, grew both in monolayer and in shaker suspension culture with doubling times of about 20 h. The cells grew easily at low serum concentrations and could even be cultured in serum-free medium supplemented with insulin and transferrin. The cultured cells were hyperdiploid, as were the cells of the xenograft. The cells grew easily in soft agar and formed tumours in athymic mice. When growing exponentially, the cells were almost unpigmented, but when grown to high density, their melanin content increased. Upon treatment with dimethyl sulphoxide (DMSO), retinoic acid and theophylline, as well as with the tumour promoter 12-O-tetradecanoyl phorbol-13-acetate (TPA), the cells showed growth inhibition and increased melanin synthesis.**

SEVERAL HUMAN CELL LINES have been established from primary and metastatic tumours obtained from patients with malignant melanomas (Romsdahl & Hsu, 1972; Liao *et al.*, 1975; Gerner *et al.*, 1975; Giovanella *et al.*, 1976). The isolated cell lines show considerable variability. Whereas some of the cell lines are particularly rich in melanin, others are amelanotic. Most of the cell lines grow in monolayer culture, but one cell line, established from the thoracic duct of a patient with metastatic melanoma, grows in suspension culture and is unable to attach to a surface (Oettgen *et al.*, 1968).

Experimental testing of the sensitivity of human cancers to cytostatic drugs is usually performed either on tumour cells cultured *in vitro* or on tumour xenografts growing in athymic mice. In this laboratory we are currently comparing drug sensitivity *in vitro* of cells obtained from xenografts with the sensitivity of the same xenografts *in vivo*. During this work a cell line with unusually high growth potential was isolated from a xenografted malignant melanoma. This cell line grows continuously and rapidly both in monolayer and in shaker suspension culture, and its characteristics are here described. To our knowledge, no melanoma cell line has previously been established from a human melanoma xenograft in athymic mice.

### MATERIALS AND METHODS

*Heterotransplantation in athymic mice.*—An inguinal metastasis from a malignant melanoma in a 52-year-old woman (E.F.) was removed in September 1977, and tissue from this tumour was cut into pieces measuring $2 \times 2 \times 2$ mm. These were transplanted s.c. into the flanks of athymic nude mice (BALB/*c*/*nu*/*nu*), purchased from Gl. Bomholt Gaard, Ry, Denmark. Tumour growth was observed after 2–3 weeks, and further transplantation was carried out every 5 weeks when the tumours had reached a diameter of $\sim$10 mm. Cells cultivated *in vitro* were scraped off the monolayer and $10^7$ cells were inoculated s.c. into athymic mice.

* Fellow of The Norwegian Cancer Society.

*Histological examination.*—Tumour material was fixed in 4% formaldehyde and 1% glutaraldehyde in phosphate buffer, and paraffin sections were stained with haematoxylin and eosin, with Fontana Masson stain for melanin and Perls' stain for iron.

*Electron microscopy.*—Solid tissue pieces were cut in cubes of 2mm sides and cultured cells spun down to pellets using low-speed centrifugation, before fixation in 4% cacodylate-buffered glutaraldehyde. Postfixation in osmium tetroxide and dehydration in graded alcohols were followed by embedding in an Epon–Araldite mixture. Semi-thin sections were cut with glass knives and stained by toluidine blue for light-microscopic examination, while ultra-thin sections were cut with diamond knives, mounted on naked copper grids and doubly stained with uranyl acetate and lead citrate before examination in the transmission electron microscope.

Coverslips with cultured cells were fixed by immersion in 4% cacodylate-buffered glutaraldehyde, dehydrated in graded alcohols and critical-point dried before sputter coating with gold and examination in the scanning electron microscope.

*Cultivation* in vitro.—A xenograft (10 mm in diameter) was removed aseptically, minced into small pieces measuring 1–2 mm, and dispersed in 10 ml serum-containing medium. The material was transferred to Falcon 3013 flasks (25 $cm^2$) and incubated at 37°C in a humidified atmosphere of 5% $CO_2$ in air. After 24 h, medium was added to give a volume of 5 ml in each flask. The cells were subcultured twice a week by treatment with 0·05% trypsin/0·02% EDTA solution for 5 min. The medium used was usually RPMI 1640 with 25mM Hepes and L-glutamine (Gibco Biocult, Glasgow, Scotland) supplemented with 15% foetal calf serum (FCS), 100 i.u./ml penicillin and 100 $\mu$g/ml streptomycin. In certain experiments Dulbecco's modified medium supplemented with 15% FCS, penicillin and streptomycin was used. When cells were grown in medium with 2% serum or in serum-free medium, non-essential amino acids and insulin (1·5 $\mu$g/ml) were added. In the case of serum-free medium transferrin (1 $\mu$g/ml) was added as well. In shaker suspension cultures cells were grown in 500ml bottles containing 200 ml RPMI 1640 medium, supplemented with 15% FCS. Dilution with fresh medium was done three times a week.

Plating efficiency of growing cells was determined by seeding out 1–5 $\times 10^2$ cells into Falcon 3002 Petri dishes (60 mm) containing 5 ml RPMI 1640 medium with 15% FCS. After 10 days colonies were counted after fixation with ethanol and staining with methylene blue.

The number of colony-forming cells in soft agar was determined according to the method described by Courtenay & Mills (1978). Both single-cell suspensions prepared from solid tumours in athymic mice and trypsinized cells from monolayer cultures were tested. After 2 weeks colonies were counted, using a Zeiss stereo-microscope.

Growth curves for cells cultured in serum-containing medium were determined by seeding out $2 \times 10^5$ trypsinized cells in 25cm$^2$ tissue-culture flasks and counting cells daily in parallel cultures. Medium was changed on Days 4, 6 and 8. Cells grown in serum-free medium were harvested by scraping, and seeded out at a density of $10^6$ per flask.

Parallel cultures with $1·5 \times 10^6$ cells grown in RPMI 1640 medium with 15% FCS in Falcon 3003 Petri dishes (100 mm) were treated with 1mM theophylline (Sigma Chemical Co., St. Louis, U.S.A.) 1·5% DMSO (Fluca AG, Buchs, Switzerland) $10^{-5}$M all-trans retinoic acid (Sigma Chemical Co.) and $10^{-7}$M TPA (Consolidated Midland Corp., New York, U.S.A.). Retinoic acid was dissolved in ethanol at a concentration of $10^{-2}$M, giving a concentration of ethanol in the medium of 0·1%, which did not affect the growth of the cells. TPA was dissolved in DMSO at a concentration of 1 mg/ml and the final concentration of DMSO in the medium was less than 0·01%. DMSO at this concentration did not affect growth. Cells were harvested by trypsinization and counted in haemacytometer on Days 0, 2, 4, 6 and 8, and medium was changed on Days 4 and 6. Protein was determined using the Bio-Rad protein assay (Bio-Rad Laboratories, Richmond, Calif., U.S.A.) and melanin was measured according to Whittaker (1963). A standard curve was prepared by dissolving known amounts of synthetic melanin (Sigma Chemical Co.) in 0·85M KOH.

*Chromosome and isoenzyme analyses.*—Chromosome and isoenzyme analyses were carried out on samples from xenografts and cells in tissue culture as previously described (Tveit *et al.*, 1980).

### RESULTS

*Original tumour and xenografts*

When grown as xenograft in athymic mice, the melanoma (E.F.), after a latent period of ~14 days, had a tumour-volume-doubling time of 6·3 days (the volume was estimated as: $\pi/6 \times$ (mean diameter$^3$). The xenografts at different passages, as well as tumours developed from cells grown in culture, showed the same histological appearance as the original tumour (Fig. 1A–C). A minority of the cells contained visible melanin granules (Fig. 1D).

*Growth in cell culture*

When pieces of a xenograft in Passage 4 (February, 1978) were seeded out into tissue-culture flasks, cells grew out from the tissue fragments in a few days. The cells were dividing rapidly and had to be subcultured after 5 days. Since then subculturing has been carried out twice a week. Throughout this period the cells have been growing rapidly without any period of slow growth. After the 70th subculture, we considered the cells as a permanent line and termed it FME. Fibroblasts which could have originated from the murine stromal cells of the xenograft have never been observed in the cultures.

FIG. 1.—Photomicrographs of the malignant melanoma. ×225. A. Patient metastasis. H. & E. B. Athymic mouse xenograft. H. & E. C. Tumour formed in athymic mouse by inoculating cultured cells. H. & E. D. Xenograft stained with Fontana Masson stain.

The FME cells are bipolar or triangular, some with processes and dendrite-like structures of various lengths (Fig. 2A). When the FME cells were grown in RPMI 1640 medium with 15% FCS the doubling time was 18 h (Fig. 3) whereas in Dulbecco's modified medium with 15% FCS the doubling time was 26 h. Cells in the 6th subculture (FM6 cells) grown in serum-supplemented RPMI 1640 medium, had a somewhat longer doubling time of 32 h. The permanent line, FME, grew easily in RPMI 1640 medium with 2% FCS and insulin (doubling time 26 h) and it could even be grown continuously in serum-free medium supplemented with insulin and transferrin. However, under the latter conditions the growth was slower, with a doubling time of ~42 h.

Several investigators have shown that the synthesis of melanin depends on cell density and proliferation rate (Silagi, 1969; Kitano & Hu, 1970; Romsdahl & Hsu, 1972). When grown in RPMI 1640 medium the FME cells contained very small amounts of melanin. Also, during exponential growth in Dulbecco's medium, the cells contained little melanin, but they became more pigmented when they reached high cell density. At the highest cell density the content of melanin per $10^6$ cells was 7 times higher than that at the lowest density (Fig. 4, control curves). The melanin content calculated per mg of cell protein was 5 times higher at the highest than at the lowest cell density.

The FME cells were easily cultivated in shaker suspension culture. Already in the 6th subculture the cells were able to grow in suspension. The doubling time for the

FIG. 2.—Photomicrographs of FME cells in monolayer culture. Phase contrast. × 200. A. Cells grown in Dulbecco's modified medium. B. Cells grown in medium with 1·5% DMSO for 2 days.

FIG. 3.—Growth curves of FME and FM6 cells in monolayer culture.

established cell line was 21 h, in RPMI 1640 medium with 15% FCS. This is only slightly longer than in monolayer culture (18 h).

Both FM6 and FME cells gave rise to tumours when $10^7$ cells were inoculated into athymic mice. The FM6 tumours had a lower growth rate than the xenografts, while the FME tumours had about the same growth rate as the xenografts.

*Plating efficiency*

The plating efficiencies in Petri dishes of cells in the 6th subculture (FM6), the 25th subculture (FM25) and of the established cell line (FME) were 53%, 96%, and 95% respectively. The corresponding values for FM6 and FME cells plated in soft agar were 27% and 78% respectively. A xenograft in Passage 14 had plating efficiency in soft agar of ~4%, while tumours formed when inoculating FM6 and FME cells into athymic mice had plating efficiencies of 14% and 55% respectively.

*Ultrastructure*

All examined cells from the original tumour, from xenografts at different passages, and from the FME cells in tissue culture, contained typical melanosomes at different stages of maturation (Fig. 5). Both the average number and the matura-

FIG. 4.—FME cells treated with 1mM theophylline (△), $10^{-5}$M retinoic acid (□), 1·5% DMSO (●) and $10^{-7}$M TPA (■) and controls (○). A. Growth curves. B. Melanin content per $10^6$ cells. C. Melanin content per mg protein.

tion range of the melanosomes were the same in cells from patient biopsy, xenografts and cell culture. In individual cells within each specimen, however, the number of melanosomes was very variable.

The cultured cells were spindle- to star-shaped and formed meshes or whirls (Fig. 6). Their surfaces were in part smooth and in part covered with microvilli and small blebs.

*Isoenzyme studies*

In cell cultures originating from human xenografts in athymic mice the possibility always exists that the multiplying cells are of murine rather than of human origin. Therefore, isoenzyme analyses were carried out both on the xenografts and on the tissue-culture cells. In each case, the same isoenzyme patterns were found in xenografts and in cultured cells. Studies of glucose 6-phosphate dehydrogenase (G6PD) revealed human Type B isoenzyme, clearly excluding the possibility of HeLa cell contamination as HeLa cells produce the Type A isoenzyme of G6PD. Lactate dehydrogenase (LDH) showed a human pattern with about equal production of A and B polypeptide chains.

*Karyology*

We also compared the chromosome counts in the xenografted tumour with early and late passage of cells *in vitro*. A histogram of chromosome counts in cells of the xenograft (Fig. 7) revealed chromosome numbers ranging from 37 to 74 with a modal number of 58. Both the FM6 and the FME metaphases had a modal chromosome number of 54, with counts ranging from 25 to 93 and 18 to 103 respectively. Banding of chromosomes in FME cells disclosed several marker chromosomes (Fig. 8).

*Effect of DMSO, TPA, retinoic acid and theophylline on cell growth and melanin synthesis*

Many melanoma cell lines have shown signs of terminal differentiation upon stimulation with certain chemicals (Kreider *et al.*, 1975; Lotan *et al.*, 1978, 1979; Huberman *et al.*, 1979) as judged by

FIG. 5.—Transmission electron micrographs. Uranyl acetate and lead citrate stain. A. Patient metastasis (×6,000). The cell in the centre contains numerous melanosomes in contrast to the cell in the lower right corner. Inset shows melanosomes at higher magnification (×28,000). B. Xenograft (×6,000). Inset shows melanosomes at higher magnification (×40,000). C. Cultured FME cells (×4,000). Some of the cells contain few melanosomes, while these organelles were numerous in others (inset, ×12,000).

morphological changes such as formation of dendrite-like processes and biochemical alterations with easily measurable changes in the production of the pigment melanin. In order to examine whether or not the FME cells could be stimulated in a similar way, we treated exponentially growing cells with theophylline, retinoic acid, DMSO and the tumour promoter TPA (Fig. 4). In untreated cultures the content of melanin increased with increasing density of cells as mentioned previously. TPA ($10^{-7}$M) had a dramatic effect on cell growth and melanin synthesis. Most of the cells developed longer and more slender dendritic processes. From Day 2 on the

### DISCUSSION

The human melanoma cell line, FME, here described, is interesting for several reasons. It grows rapidly both in monolayer and shaker suspension culture (doubling time less than 24 h), it has low serum requirements and can even be grown without serum, provided insulin and transferrin were present. Furthermore, the

FIG. 8.—Karyotype of a metaphase of cultured FME cells. Trypsin-Giemsa banding method. Number of chromosomes: 52. Inset shows 4 marker chromosomes. p: short arm, q: long arm.

cell line synthesizes melanin in amounts which depend on the proliferation rate, and growth inhibition and increased melanin synthesis can be induced by different agents.

Cell lines are in most cases established from human tumour biopsy material by spontaneous emergence of rapidly dividing cells in a culture previously growing slowly. However, the FME cells, established from a xenograft in athymic mouse, grew rapidly already from the start of the culture and reduction in growth rate was never observed. This rapid growth *in vitro* could possibly be explained by selection in the xenograft of tumour cells with high growth potential. However, in other cases cells from human xenografts in athymic mice, including melanomas, carcinomas and sarcomas, did not grow easily in tissue culture.

Permanent tumour cell lines have often been supposed to maintain the biological characteristics of the *in situ* tumour cells from which the cell lines were initiated. However, the morphological, biochemical and cytogenetic properties as well as the chemosensitivity of tumour cells under various *in vivo* and *in vitro* conditions are inadequately examined. Cells from the permanent melanoma cell line here described (FME) as well as cells in an early subculture (FM6) showed the same ultrastructure as the original patient tumour and the xenografts. FM6 and FME cells had equal modal chromosome numbers (54) slightly less than cells from a xenograft (58), and the isoenzyme patterns of xenografts and cells in tissue culture were identical. Growth rate and plating efficiency were, however, higher for the FME cells in culture and FME tumours in athymic mice than for the FM6 cells and FM6 tumours. Cells from the xenografts had even lower plating efficiency than cells from the FM6 tumours, but the growth rate was higher and equal to that of the FME tumours. Preliminary studies on the *in vitro* chemosensitivity of the FM6 and FME cells, the FM6 and FME tumours as well as the xenografts, indicate that for some of the drugs the sensitivity is constant, but for others it differs to some extent. The tendency is for the tumour cells to become more resistant when grown *in vitro*. So it appears that some of the biological characteristics of the melanoma cells were maintained, while others were changed when tumour cells from a xenograft were cultivated continuously in tissue culture. Thus, extrapolation of data obtained on cell lines in tissue culture to the tumours from which they originated should be made with great caution.

In the cell line here described chromosome and isoenzyme analyses did not reveal any contamination with mouse stromal cells. This is in clear contrast to the results obtained by cultivation of cells from a human embryonal carcinoma xenograft (Tveit *et al.*, 1980) and from another human melanoma xenograft (unpublished) where abnormal mouse cells appeared in the culture and were growing rapidly along with the human tumour cells.

Several cell lines have been cultivated in serum-free medium supplemented with hormones and growth factors (Hayashi & Sato, 1976; Barnes & Sato, 1979). A mouse cell line derived from B16 melanoma grew indefinitely in medium supplemented with insulin, transferrin, testosterone, follicle-stimulating hormone, nerve growth factor and luteinizing hormone (Mather & Sato, 1979). Also the FME cells could be grown continuously in serum-free RPMI 1640 medium, supplemented only with insulin, transferrin and non-essential amino acids.

Melanin production may be influenced by several chemicals. Thus, we have shown here that both growth inhibition and increased melanin synthesis was induced in the FME cell line upon treatment with theophylline, retinoic acid, DMSO and the tumour promoter TPA. These compounds have been shown to induce differentiation in a variety of cells. Thus, the mouse B16 melanoma showed growth inhibition and increased melanin production when treated with theophylline (Kreider *et al.*, 1975). Retinoic acid induced differentiation of embryonal carcinoma cells into endoderm (Strickland & Mahdavi, 1978) and inhibited growth of murine and human melanoma cell lines (Lotan *et al.*, 1978, 1979). DMSO induced differentiation of mouse and human leukaemia cells (Friend *et al.*, 1971; Collins *et al.*, 1978) and of a human melanoma cell line (Huberman *et al.*, 1979). Phorbol esters (mouse skin tumour promoters), however, have opposite effects in different cell lines. Thus, these agents inhibited differentiation of several avian and murine cells, but induced differentiation in human myeloid leukaemia cells and of a human melanoma cell line (Huberman & Callaham, 1979; Huberman *et al.*, 1979). Our findings of growth inhibition and increased melanin synthesis of the human FME cells upon stimulation with TPA support the view that human cells respond differently from cells of other species to phorbol esters.

The high growth potential and the response to agents known to induce terminal differentiation in certain other cell lines makes the FME cell line useful in studies on factors controlling growth and differentiation.

This work was supported by The Norwegian Cancer Society. The authors are indebted to Professor A. Pihl for advice and discussion, to Dr A. Brøgger for help with the chromosome banding and to Dr K. Eiklid for help with the isoenzyme analyses. The technical assistance of Mrs J. Jacobsen is gratefully acknowledged.

### REFERENCES

BARNES, D. & SATO, G. (1979) Growth of a human mammary tumour cell line in a serum-free medium. *Nature*, **281**, 388.

COLLINS, S. J., RUSCETTI, F. W., GALLAGHER, R. E. & GALLO, R. C. (1978) Terminal differentiation of human promyelocytic leukemia cells induced by dimethyl sulfoxide and other polar compounds. *Proc. Natl Acad. Sci. U.S.A.*, **75**, 2458.

COURTENAY, V. D. & MILLS, J. (1978) An *in vitro* colony assay for human tumours in immune-suppressed mice and treated *in vivo* with cytostatic agents. *Br. J. Cancer*, **37**, 261.

FRIEND, C., SCHER, W., HOLLAND, J. G. & SATO, T. (1971) Hemoglobin synthesis in murine virus-induced leukemic cells *in vitro*: Stimulation of erythroid differentiation by dimethyl sulfoxide. *Proc. Natl Acad. Sci. U.S.A.*, **68**, 378.

GERNER, R. E., KITAMURA, H. & MOORE, G. E. (1975) Studies of tumor cell lines derived from patients with malignant melanoma. *Oncology*, **31**, 31.

GIOVANELLA, B. C., STEHLIN, J. S., SANTAMARIA, C. & 6 others (1976) Human neoplastic and normal cells in tissue culture. I. Cell lines derived from malignant melanomas and normal melanocytes. *J. Natl Cancer Inst.*, **56**, 1131.

HAYASHI, I. & SATO, G. H. (1976) Replacement of serum by hormones permits growth of cells in a defined medium. *Nature*, **259**, 132.

HUBERMAN, E. & CALLAHAM, M. F. (1979) Induction of terminal differentiation in human promyelocytic leukemia cells by tumor-promoting agents. *Proc. Natl Acad. Sci. U.S.A.*, **76**, 1293.

HUBERMAN, E., HECKMAN, C. & LANGENBACH, R. (1979) Stimulation of differentiated functions in human melanoma cells by tumor-promoting agents and dimethyl sulfoxide. *Cancer Res.*, **39**, 2618.

KITANO, Y. & HU, F. (1970) Proliferation and differentiation of pigment cells *in vitro*. *J. Invest. Dermatol.*, **55**, 444.

KREIDER, J. W., WADE, D. R., ROSENTHAL, M. & DENSLEY, T. (1975) Maturation and differentiation of B 16 melanoma cells induced by theophylline treatment. *J. Natl Cancer Inst.*, **54**, 1457.

LIAO, S. K., DENT, P. B. & MCCULLOCH, P. B. (1975) Characterization of human malignant melanoma cell lines. I. Morphology and growth characteristics in culture. *J. Natl Cancer Inst.*, **54**, 1037.

LOTAN, R. (1979) Different susceptibilities of human melanoma and breast carcinoma cell lines to retinoic acid-induced growth inhibition. *Cancer Res.*, **39**, 1014.

LOTAN, R., GIOTTA, G., NORK, E. & NICOLSON, G. L. (1978) Characterization of the inhibitory effects of retinoids on the *in vitro* growth of two malignant murine melanomas. *J. Natl Cancer Inst.*, **60**, 1035.

MATHER, J. P. & SATO, G. H. (1979) The growth of mouse melanoma cells in hormone-supplemented, serum-free medium. *Exp. Cell Res.*, **120**, 191.

OETTGEN, H. F., AOKI, T., OLD, L. J., BOYSE, E. A., DE HARVEN, E. & MILLS, G. M. (1968) Suspension culture of a pigment-producing cell line derived from a human malignant melanoma. *J. Natl Cancer Inst.*, **41**, 827.

ROMSDAHL, M. M. & HSU, T. C. (1972) Establishment and characterization of human malignant melanoma cell lines grown *in vitro*. In *Pigmentation. Its genesis and biologic control*. Ed. Riley. New York: Appleton-Century-Crofts. p. 461.

SILAGI, S. (1969) Control of pigment production in mouse melanoma cells *in vitro*. Evocation and maintenance. *J. Cell Biol.*, **43**, 263.

STRICKLAND, S. & MAHDAVI, V. (1978) The induction of differentiation in teratocarcinoma stem cells by retinoic acid. *Cell*, **15**, 393.

TVEIT, K. M., FODSTAD, Ø., BROGGER, A. & OLSNES, S. (1980) Human embryonal carcinoma grown in athymic mice and *in vitro*. *Cancer Res.*, **40**, 949.

WHITTAKER, J. R. (1963) Changes in melanogenesis during the dedifferentiation of chick retinal pigment cells in cell culture. *Dev. Biol.*, **8**, 99.